# Large‐ and small‐scale geographic structures affecting genetic patterns across populations of an Alpine butterfly

**DOI:** 10.1002/ece3.8157

**Published:** 2021-09-28

**Authors:** Daronja Trense, Ary A. Hoffmann, Klaus Fischer

**Affiliations:** ^1^ Institute for Integrated Natural Sciences, Zoology University Koblenz‐Landau Koblenz Germany; ^2^ Pest & Environmental Adaptation Research Group School of Biosciences Bio21 Institute Parkville Vic. Australia

**Keywords:** barrier to dispersal, evolutionary significant unit, genetic differentiation, genetic diversity, glacial refuges, SNP outlier loci

## Abstract

Understanding factors influencing patterns of genetic diversity and the population genetic structure of species is of particular importance in the current era of global climate change and habitat loss. These factors include the evolutionary history of a species as well as heterogeneity in the environment it occupies, which in turn can change across time. Most studies investigating spatio‐temporal genetic patterns have focused on patterns across wide geographic areas rather than local variation, but the latter can nevertheless be important particularly in topographically complex areas. Here, we consider these issues in the Sooty Copper butterfly (*Lycaena tityrus*) from the European Alps, using genome‐wide SNPs identified through RADseq. We found strong genetic differentiation within the Alps with four genetic clusters, indicating western, central, and eastern refuges, and a strong reduction of genetic diversity from west to east. This reduction in diversity may suggest that the southwestern refuge was the largest one in comparison to other refuges. Also, the high genetic diversity in the west may result from (a) admixture of different western refuges, (b) more recent demographic changes, or (c) introgression of lowland *L. tityrus* populations. At small spatial scales, populations were structured by several landscape features and especially by high mountain ridges and large river valleys. We detected 36 outlier loci likely under altitudinal selection, including several loci related to membranes and cellular processes. We suggest that efforts to preserve alpine *L. tityrus* should focus on the genetically diverse populations in the western Alps, and that the dolomite populations should be treated as genetically distinct management units, since they appear to be currently more threatened than others. This study demonstrates the usefulness of SNP‐based approaches for understanding patterns of genetic diversity, gene flow, and selection in a region that is expected to be particularly vulnerable to climate change.

## Graphical abstract

We aim at unraveling the spatial and historical factors having shaped the contemporary genetic structure of the butterfly *Lycaena tityrus* in the European Alps at different spatial scales. SNP analyses showed a strong genetic differentiation between populations in the European Alps, a decrease in genetic diversity from west to east, and a clear separation of the dolomite populations. At a smaller scale, high mountain ridges and large river valleys hampered gene flow and structured populations.

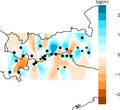

## 1 INTRODUCTION

An important aim in contemporary evolutionary biology and ecology is to understand the dynamics of genetic diversity and structure of a range of representative species in space and time, based on demographic history and geographic factors in the environment (Després et al., 2019; Jackson et al., 2018). Understanding such spatial and temporal variation is of particular relevance in the current era of dramatic climate change and habitat loss (Butchart et al., 2010; Lucena‐Perez et al., 2020; Wilson et al., 2016). Spatio‐temporal features form biogeographic patterns such as through barriers or corridors for dispersal, affecting the ability of organisms to locate suitable habitat and expand their ranges (Hewitt, 2003; McRae & Beier, 2007; Sheth et al., 2020; Zhivotovsky, 2016). However, the impacts of such features are often species‐specific, depending on the ability of species to disperse and adapt, and on the scale and extent of (un‐)suitable habitat and barriers (Hewitt, 1999; Keyghobadi, 2007; Sheth et al., 2020).

On a temporal scale, past climates influence the present‐day distribution of species (Parmesan et al., 1999; Schmitt et al., 2006; Thomas & Lennon, 1999). In Europe, populations of many species have been strongly affected by oscillations of cold and warm periods in the Pleistocene, which have resulted in latitudinal and altitudinal range shifts (Coope, 1970; Hewitt, 1996, 1999; Schmitt et al., 2006). During cold periods, many European species became restricted to southern refuges from which they expanded northwards in warm periods (Després et al., 2019; Strandberg et al., 2011; Tab erlet et al., 1998). On a spatial scale, environmental heterogeneity is the main factor influencing the population structure of species (Kokko & López‐Sepulcre, 2006; Lowe & McPeek, 2014; Yang et al., 2017). Heterogeneous environments, characterized by topographic barriers, landscape clines, or unsuitable habitats, affect population connectivity and the distribution of species (Jackson et al., 2018; Lowe & McPeek, 2014; Manel et al., 2003).

Geographic structures at the spatial scale can be further divided into large‐ and small‐scale structures. Small‐scale structures that might influence local or regional patterns of diversity comprise natural barriers like rivers, lakes, mountain ridges, gorges, and forests, as well as anthropogenic barriers like roads, railways, agricultural plots, and settlements (e.g., Heidinger et al., 2013; Miles et al., 2019; Nilsson et al., 2013; Sheth et al., 2020; Storfer et al., 2010). Large‐scale structures include mountain ranges, oceans, and deserts. In Europe, the Alps and the Pyrenees have formed, since the Last Glacial Maximum, large‐scale geographic barriers that are difficult to cross for less mobile species (Hewitt, 1996, 1999; Tab erlet et al., 1998). These mountain ranges currently harbor hybrid zones and endemic taxa across heterogeneous landscapes composed of unsuitable and suitable habitats in close proximity, created by small‐scale geographic features (Dagnino et al., 2020; Dirnböck et al., 2011; Hewitt, 2004; Martin et al., 2002). They provide a useful region for investigating the impact of geographic features on population structure and connectivity. Furthermore, alpine environments involve steep gradients in environmental conditions such as temperature, oxygen concentration, and ultraviolet radiation, affecting species assemblages (Cheviron & Brumfield, 2012; Montero‐Mendieta et al., 2019) including insects and their host plants (Hodkinson, 2005; Horn et al., 2006; Montero‐Mendieta et al., 2019). Thus, specific adaptations to high‐altitude environments can be expected (Cheviron & Brumfield, 2012; Karl et al., 2008, 2009; Polato et al., 2017).

Our focus here is on the way these various structures have influenced the population genetic structure and patterns of genetic variation. Most research on spatio‐temporal patterns has focused on ecosystems, ecological communities, and species distributions and much more rarely on genetic diversity within species (Coates et al., 2018; Hoban et al., 2020; Miraldo et al., 2016). A consideration of genetic diversity and population structure is important for several reasons. First, reduced genetic diversity may interfere with a species’ ability to respond to changing environments (Willi et al., 2006); it may also be a signature of a persistently small population size that can result in inbreeding (and hence inbreeding depression) in a population (Bijlsma & Loeschcke, 2012; Day et al., 2003; Frankham, 1995; Keller & Waller, 2002). Second, genetically distinct populations may be useful for delineating management units for conservation purposes (Coates et al., 2018; Moritz, 1994). If populations of a given species are sufficiently differentiated from other populations, they may form evolutionary significant units (ESUs; Frankham, 2010; Moritz, 1994; Ryder, 1986) that have different evolutionary trajectories, although this requires some caution because genetic drift can also result in genetically unique populations with limited evolutionary capacity (Weeks et al., 2016).

**FIGURE 1:**
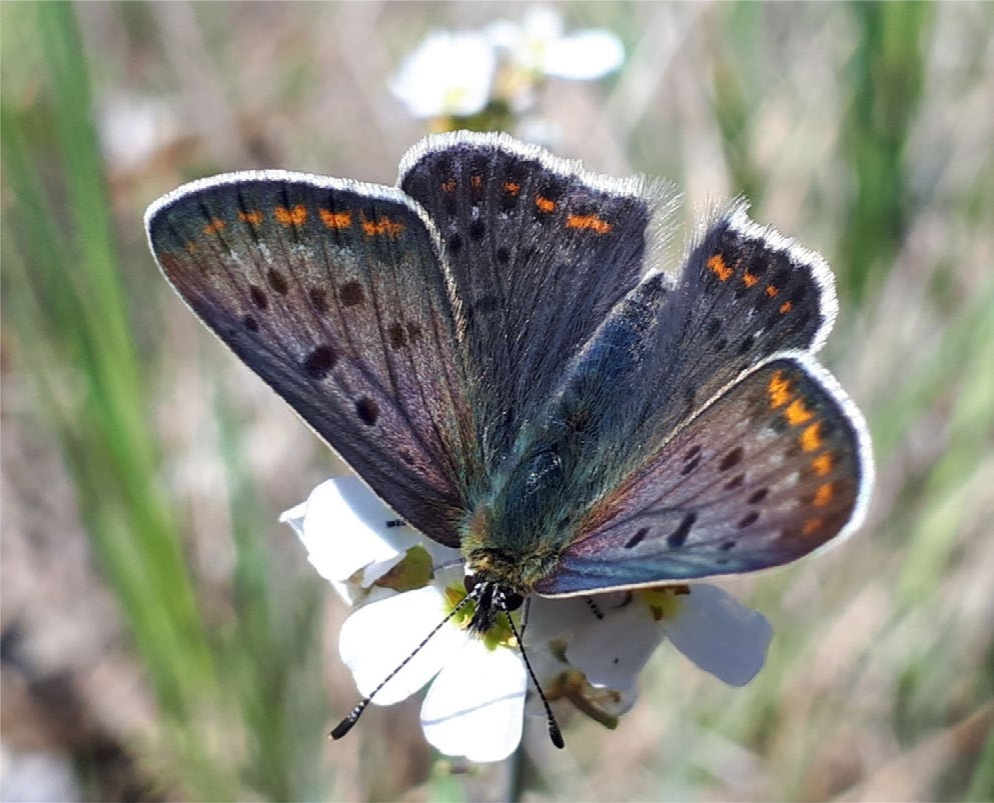
Photograph of a male Sooty Copper (*Lycaena tityrus*)

Here, we examined population genetic structure and diversity of the Sooty Copper butterfly *Lycaena tityrus* (Poda, 1761; Figure 1) in the European Alps. Butterflies are generally capable of short‐ and long‐distance dispersal, depending on landscape heterogeneity (Martin et al., 2002). *Lycaena tityrus* forms closed populations and is likely to have a limited dispersal ability which is strongly affected by dispersal barriers at small scales (Trense et al., 2021). Until recently, genetic variation in butterfly populations has been investigated through markers such as allozymes, microsatellites, or a small number of sequenced mitochondrial and nuclear genes, but inferences based on these markers have been limited by the fact that they sample only a small part of the genome (e.g., Maresova et al., 2019; Schmitt et al., 2006; Ugelvig et al., 2012). New molecular techniques now provide thousands of SNP markers across the genome allowing the identification of past refuges, genetic diversity, geographic barriers, and gene flow in butterflies with unprecedented rigor (Fountain et al., 2018; Nève, 2009).

In this study, we used genome‐wide single nucleotide polymorphisms (SNPs) to investigate genetic diversity and geographic population structure of *L. tityrus* across the eastern European Alps. In a previous study using the same species, we found high gene flow within a single Alpine valley, but also genetic structuring linked to ravines, forests, roads, and altitude (Trense et al., 2021). Here, we analyzed a much broader spatial scale by sampling *L. tityrus* individuals in 30 different valleys in the European Alps, while the previous study was confined to only one valley. In the present study, we test whether (a) populations are structured by the presence of high mountain ridges and/or large river valleys potentially comprising barriers to dispersal, (b) current patterns of genetic structure are influenced by long‐term processes such as postglacial range expansions, (c) different evolutionary lineages are present within this species, and also whether (d) populations from different altitudes show signatures of local adaption based on an analysis of outlier loci.

## 2 METHODS

### 2.1 Study organism and population sampling

**FIGURE 2:**
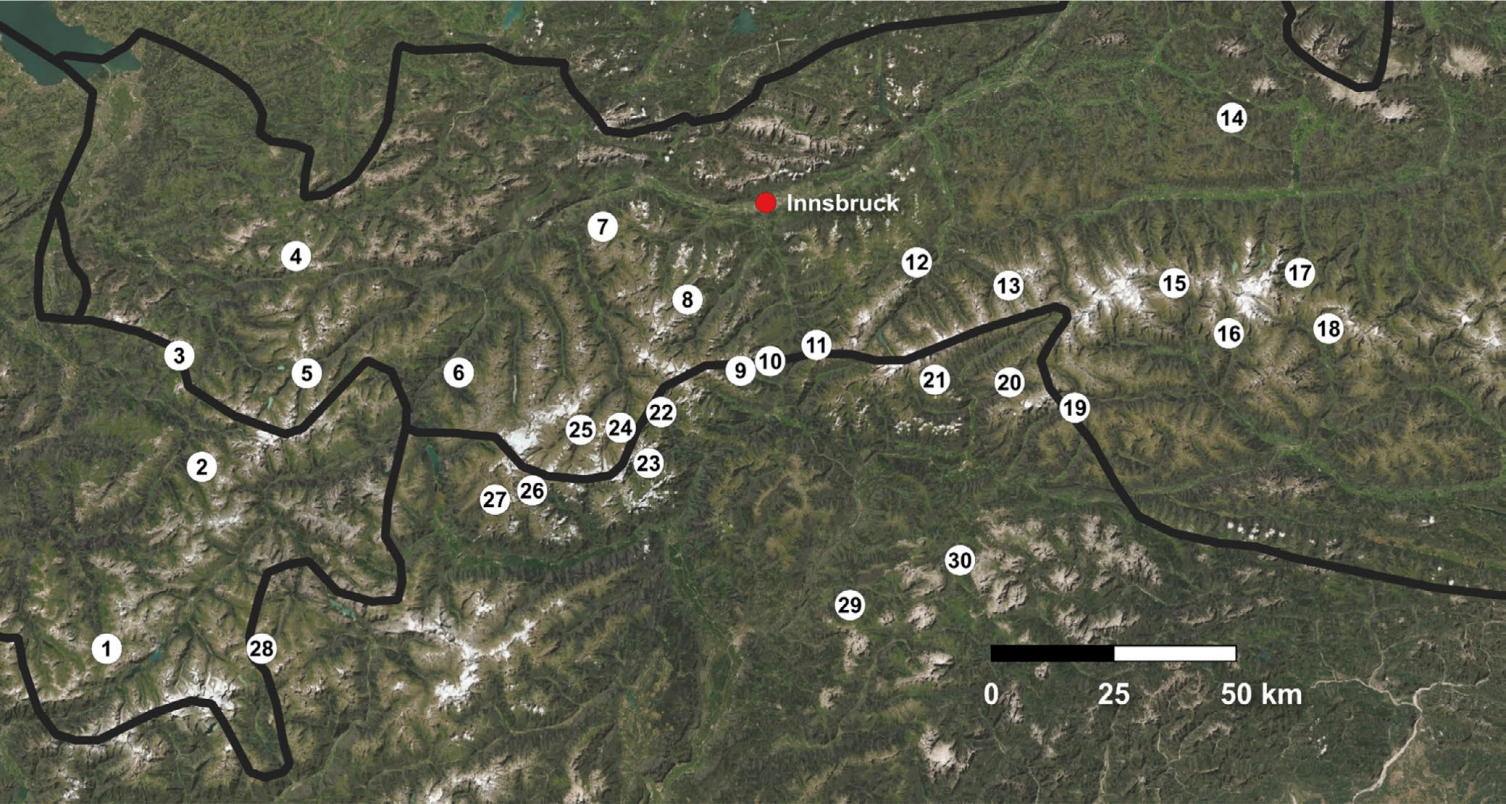
Geographic distribution of the 30 sampling locations for *Lycaena tityrus* across the European Alps. Details are given in Table 1. The black lines indicate the national borders. The map was generated with QGIS version 3.14 (www.qgis.org)

Here, we used the alpine subspecies *L. tityrus subalpinus* (Lepidoptera: Lycaenidae). *Lycaena tityrus* is a widespread butterfly of the temperate zone with a range from Western Europe to central Asia (Ebert & Rennwald, 1991). It inhabits different kinds of grassland, including moist and dry meadows, sandy heathland, bogs, and open woodland (Settele et al., 2008). The principal larval host plant is *Rumex acetosa* L., but some congeneric plant species (e.g., *Rumex acetosella* L., *Rumex scutatus* L.; Ebert & Rennwald, 1991, Settele et al., 2008, Tolman & Lewington, 2008) are also used. *Lycaena t. subalpinus* is confined to higher altitudes of the European Alps and some other mountain ranges, where it is relatively widespread and has one generation a year (Tolman & Lewington, 2008). The altitudinal distribution of *L. t. subalpinus* ranges from 1,200 to 2,500 m a.s.l. (Tolman & Lewington, 2008). Thirty populations were sampled in Austria (Salzburg, Tyrol, Vorarlberg), Italy (South Tirol, Lombardy), and Switzerland (Graubünden), spanning an altitude from ca. 1,260 to 2,110 m a.s.l. (Figure 2, Table 1). We caught nine males per population in the summers of 2018 and 2019. All 270 individuals used in the current analysis were stored in liquid nitrogen until DNA extraction. Here, we re‐used the extracted DNA of 23 from the 186 individuals tested in Trense et al. (2021). However, sequencing of these samples was done again together with the new samples.

**TABLE 1:** Sampling localities with country (Austria (A), Switzerland (CH), and Italy (I)), coordinates, altitude, and time of sampling for 30 *Lycaena tityrus* populations

No	Locality	Country	Coordinates (°N, °E)	Altitude in m	Time of sampling
1	St. Moritz	CH	46.46, 9.66	1,830	July 2019
2	Davos	CH	46.79, 9.91	1,900	July 2019
3	St. Antönien	CH	47.00, 9.85	1,670	July 2019
4	Zürs	A	47.17, 10.16	1,710	July 2019
5	Galtür	A	46.96, 10.19	1,620	July 2019
6	Pfunds	A	46.96, 10.59	1,600	June 2019
7	Kühtai	A	47.23, 10.97	1,720	June 2019
8	Stubai	A	47.10, 11.20	1,710	June 2019
9	Pflersch	A	46.97, 11.34	1,260	June 2018
10	Obernberg	A	46.98, 11.42	1,780	July 2018
11	Venntal	A	47.01, 11.54	1,540	July 2018
12	Penken	A	47.16, 11.80	1,800	July 2019
13	Zillergrund	A	47.12, 12.05	1,460	July 2019
14	Schwarzleo	A	47.42, 12.64	1,400	July 2019
15	Innergschlöss	A	47.12, 12.48	1,530	July 2019
16	Kals	A	47.03, 12.63	1,810	July 2019
17	Ferleiten	A	47.14, 12.82	1,650	July 2019
18	Heiligenblut	A	47.04, 12.89	1,750	July 2019
19	Staller Alm	A	46.90, 12.22	1,960	July 2019
20	Reintal	I	46.95, 12.05	1,790	June 2019
21	Weißenbach	I	46.95, 11.85	1,430	July 2019
22	St. Leonhard	I	46.89, 11.13	1,800	July 2018
23	Pfelders	I	46.80, 11.09	1,590	July 2018
24	Obergurgl	A	46.86, 11.02	1,930	June 2018
25	Vent	A	46.86, 10.91	1,890	June 2018
26	Schnals	I	46.75, 10.79	1,950	July 2018
27	Glieshof	I	46.73, 10.69	1,900	July 2019
28	Livigno	I	46.46, 10.07	2,100	July 2019
29	Seis	I	46.54, 11.63	1,830	June 2019
30	Amentara	I	46.62, 11.92	1,680	June 2019

Note

Numbers refer to the localities depicted in Figure 2.

### 2.2 ddRAD library preparation

From each male, we used head and thorax for the extraction of genomic DNA with the E.Z.N.A.^®^ Tissue DNA Kit (Omega Bio‐tek). We followed the manufacturers' instructions but included an additional step of RNase A treatment. Afterward, we applied double‐digest restriction site‐associated DNA sequencing (ddRADseq) following Trense et al. (2021) and used the restriction enzymes NlaIII and MluCI. We pooled individuals into four libraries, each containing DNA fragments from individuals with different adapter pairs. Libraries were cleaned with 1.5× volume of Sera‐Mag beads. We selected 250–400 base pair (bp) fragments using a 2% gel cassette and Pippin‐Prep software 4.3 (Sage Science). This was followed by a polymerase chain reaction (PCR) enrichment in a 10‐µl reaction with 1 µl of size selected DNA, 2 µl 5× Phusion^®^ HF Reaction Buffer, 0.2 µl dNTPs (10 mM), 0.1 µl (100 units) Phusion® HF DNA Polymerase (New England BioLabs Inc.), nuclease‐free water, and 2 µl (10 µM) each of Illumina P1 and P2 primers (Peterson et al., 2012). The profile of thermal cycling consisted of denaturation at 98°C for 30 s, followed by 12 cycles with 10 s at 98°C, 30 s at 65°C, and 70 s at 72°C, and an extension for 5 min at 72°C. For the final library, we used seven PCRs. The four libraries were sequenced on an Illumina NovaSeq 6000 platform generating 150‐bp paired‐end reads.

### 2.3 SNP calling

For calling SNPs, we used a de novo pipeline without a reference genome, namely, the *process_radtags* program within stacks version 2.3 (Catchen et al., 2011, 2013). Here, we demultiplexed the sequence data by separating the individuals through their unique barcode, removed adapter sequences, trimmed the reads to 120 bp length, and discarded low‐quality reads (Phred score < 20). The number of loci was 2,553,678. Afterward, we ran the stacks *denovo_map* pipeline using one individual per sampled population to generate a catalogue of loci by treating all individuals as belonging to one population. Each of the 270 individuals was then matched against this catalogue, and SNPs were subsequently called. We filtered the data to retain SNPs, which were present in more than 50% of the 270 individuals, and then retained the first SNP in each ddRAD locus. Note that some ddRAD loci can be located in the same gene. Further filtering with the program vcftools version 0.1.11 (Danecek et al., 2011) was done to retain loci that were at Hardy–Weinberg equilibrium, had a minor allele count of >2 (Linck & Battey, 2019), missing data of <5%, and a mean depth of 20–45. For filtering, individuals of the same sampling location were treated as belonging to one population but excluding two individuals of population 9 because of incomplete sequencing. The final dataset contained 13,455 SNPs from a total of 99,717 polymorphic RAD loci.

### 2.4 Analysis of relatedness

To avoid biased population analyses, we used the *Triadic Likelihood Estimator* (TrioML, giving a relatedness coefficient) in coancestry version 1.0.1.9 (Wang, 2007, 2011) to calculate the pairwise relatedness among individuals. Individuals with a TrioML estimator of ≥0.250 are most likely full‐siblings and between 0.125 and 0.249 probably half‐siblings. Real ancestry cannot be assessed by TrioML though, but only genetic relatedness. We found several related individuals including seven probable pairs of full‐siblings. One full‐sibling per probable pair was excluded from downstream analyses. To confirm putative full‐ and half‐siblings, we additionally used the *Specific Hypothesis Test* function with 9,999 permutations implemented in the program ml‐relate (Kalinowski et al., 2006).

### 2.5 Isolation by distance and resistance

To test for isolation by distance, we calculated pairwise genetic distances between individuals according to Bray Curtis (Bray & Curtis, 1957), using the package “vegan” (Oksanen et al., 2019) in r version 3.5.2 (R Core Team, 2015). Pairwise, individual‐based geographic distances were calculated as (a) Euclidean distances with the function *PointDistance* (plane) in the package “raster” (Hijmans & van Etten, 2016) in R, based on the UTM system, and as (b) altitudinal distances by a distance matrix. We tested for correlations between genetic and geographic distance (isolation by distance) as well as altitudinal distance by using Mantel tests (using the *Mantel* function from the “vegan” package in r; Mantel, 1967) with 9,999 permutations. Furthermore, we used an *estimated effective migration surface* (eems; Petkova et al., 2016) algorithm to visualize geographic regions that deviate from isolation by distance, thus representing dispersal corridors or barriers, and that show high or low genetic diversity. We tested the deme numbers 200 and 600 using *RunEEMS_SNPS* in eems with three independent runs of 200,000 Markov Chain Monte Carlo (MCMC) iterations, each with a burn‐in of 100,000 and a thinning interval of 9,999.

To further analyze isolation by resistance (IBR), we first modeled the distribution of the 30 *L. tityrus* populations using Maxent version 3.4.1 (Phillips et al., 2017) by including several landscape features, namely, altitude, grass cover, roads, slope, tree cover, and water bodies. The raster file data for altitude were taken from Natural Earth (naturalearthdata.com), while the data for grass cover, tree cover, and water bodies were taken from Copernicus (land.copernicus.eu), and for roads from the Global Roads Inventory Project (GRIP) dataset of the global biodiversity model for policy support (globio.info). We used the digital surface model from Copernicus to generate the slopes using the function Slope in the Raster‐analysis in QGIS version 3.14 (www.qgis.org). The packages “raster” and “rgdal” in R were used for identical landscape features in resolution, extent, and projection. For Maxent distribution modeling, we selected default settings but changed the random test percentage to 25. We used the reverse suitability values of Maxent estimates for the landscape features (altitude, grass cover, roads, slope, tree cover, and water bodies). We then ran circuitscape version 4.0 (McRae et al., 2013) to receive resistance surfaces and distances for these features and followed Cushman et al. (2006) to analyze the relationship between genetic and landscape distance matrices by using the simple and partial Mantel test in the package “ecodist” (Goslee & Urban, 2007) in R with 9,999 permutations. We performed five partial Mantel tests for each landscape feature to analyze the relationships between genetic and landscape distance matrices, partialling out the effect of other landscape distance matrices. To account for the nonindependency of records from one individual, we additionally ran maximum‐likelihood population effects (MLPE) following Clarke et al. (2002) and Peterman (2018).

### 2.6 Analysis of molecular variance (AMOVA)

We used an hierarchical AMOVA in arlequin version 3.5 (Excoffier & Lischer, 2010) to assess the genetic variance among the 30 populations, among individuals within these populations and within individuals.

### 2.7 Analysis of genetic structure

We calculated observed and expected heterozygosity, inbreeding coefficient, and global *F*_ST_ for all 30 populations using the packages “adegenet” (Jombart, 2008; Jombart & Ahmed, 2011) and “hierfstat” (Goudet, 2005) in r. The pairwise *F*_ST_ values were calculated in arlequin. Furthermore, we identified genetic clusters using the function *SNMF* (sparse non‐negative matrix factorization) in the package “LEA” (Frichot & François, 2015) in R. We tested 31 ancestral populations (*K* = 1–31) with 100 repetitions for each *K*. The minimal cross‐entropy value was selected to visualize the genetic clustering. Based on the results of the eems analysis which indicated that major river valleys and high mountain ridges may comprise genetic barriers for this species (cf. Table 2), as well as visual inspection of aerial maps, we grouped the individuals according to different factors: (a) the 30 populations (sample locations), that is, each sampling location was treated as a separate group; (b) 13 groups separated by major river valleys (the groups included the following populations: 1, 4, 14, 28, 2 + 3 + 5, 6 + 23–27, 7 + 8, 9 + 10 + 22, 11 + 12, 13 + 21, 15–18, 19 + 20, and 29 + 30); (c) 18 groups separated by major river valleys and/or high mountain ridges, consisting of the populations 2, 4, 6, 7, 8, 14, 19, 1 + 28, 3 + 5, 9–11, 12 + 13, 15 + 16, 17 + 18, 20 + 21, 22 + 23, 24 + 25, 26 + 27, and 29 + 30; and (d) 15 groups separated by high mountain ridges, consisting of populations 1, 2, 4, 6, 7, 19, 28, 3 + 5, 8–11 + 22 + 23, 12 + 13, 14 + 17 + 18, 15 + 16, 20 + 21 + 29 + 30, 24 + 25, and 26 + 27. We calculated pairwise genetic distances on an individual basis (Bray & Curtis, 1957). To test how well the different groups were statistically supported, we ran distance‐based redundancy analyses (dbRDA; Legendre & Anderson, 1999) with the r package “vegan” (Oksanen et al., 2007).

**TABLE 2:** Results of distance‐based redundancy analyses for the effects of different factors on the population genetic structure of *Lycaena tityrus* in the European Alps

Variable	*df*	*SS*	*F*	*η^2^*	*p*
Populations	29	0.372	2.319	0.226	**<.0001**
River valleys	12	0.230	3.342	0.139	**<.0001**
River valleys & mountains	17	0.281	2.930	0.170	**<.0001**
High mountain ridges	14	0.251	3.154	0.152	**<.0001**

Note

Factors used are the sampled locations (populations) as well as different clusters as defined by major river valleys, major river valleys in combination with high mountain ridges, and high mountain ridges. Effect sizes are given as partial Eta squared (*η^2^*), and significant *p*‐values are given in bold.

Abbreviations: *df*, degrees of freedom; *F*, *F*‐value; *p*, error probability; ss, sum of squares; *η^2^*, Eta squared.

For analyzing the population demographic history, we used the approximate Bayesian computation (ABC) method as implanted in diyabc version 2.1.0 (Cornuet et al., 2014). Based on the results of the *SNMF* analysis, four population clusters (western, central, eastern, and southeastern populations) were defined (Figure 5b). For the four population clusters, we ran seven scenarios (Figure S1.1, Appendix S1). We set the conditions of the prior time distributions to t1 < t2 to avoid incongruences in the simulated genealogies. The overall performance of scenarios was assessed with a principal component analysis using 100,000 simulated datasets and the observed data. We then assessed the posterior probability of the scenarios with a logistic regression procedure based on the 1% closest simulated datasets compared to the observed data. We used the posterior distributions for the best supported scenario for simulating 1,000 pseudo‐observed datasets to assess whether this model could successfully reproduce the observed data.

### 2.8 Outlier loci analysis

To identify potential outlier loci linked to altitude, we used the altitude of each sampling location; that is, the individuals of one location were assigned the same altitude. Three different *F*_ST_ outlier analyses were performed to minimize the risk of false positives, namely, baypass version 2.1 (Gautier, 2015), FDIST2 in arlequin (Excoffier & Lischer, 2010), and bayescan version 2.1 (Foll & Gaggiotti, 2008). The program baypass calculates an *F*_ST_ analogue called XtX and can perform association tests between genomic outliers and population‐specific covariables, such as in this case altitude. FDIST2 in arlequin finds all loci under selection based on the patterns of genetic diversity found in a population. bayescan identifies loci under selection using differences in allele frequencies and considers the uncertainty of allele frequencies due to small sample sizes. Furthermore, we ran *LFMM* (latent factor mixed models) in the package “LEA” in R to find outlier SNPs linked to altitude and different temperature variables (mean temperature, maximal temperature of the warmest month, mean temperature of the wettest and warmest quarter, and mean temperature of the driest and coldest quarter). We controlled for a false discovery rate in baypass, bayescan, and *lfmm*. SNPs were considered as potential outliers if the *p*‐values were below 0.05, *F*_ST_ were above 0.1, and at least two out of the three outlier analyses identified them as SNPs under selection. We searched for the sequences of the respective SNPs and corresponding protein sequences in the genome annotation of *Calycopis cecropis* (Lepidoptera: Lycaenidae) v. 1.1 using samtools version 1.2 (Li, 2011; Li et al., 2009). For characterization of loci under selection, we used omicsbox version 1.3.11 (BioBam Bioinformatics, 2019 https://www.biobam.com/omicsbox) for functional analysis of the identified proteins.

## 3 RESULTS

### 3.1 Analysis of relatedness

Comparisons of relatedness across all 268 individuals revealed seven probable pairs of full‐siblings (0.607 < TrioML < 0.291) and 114 probable pairs of half‐siblings (0.246 < TrioML < 0.126). The program ml‐relate confirmed all putative pairs of full‐ and half‐siblings. All pairs of full‐siblings were found within the same sampling location. In four out of the 114 putative pairs of half‐siblings, the half‐siblings were found in different populations (all in populations 29 and 30), separated by a geographic distance of 24 km and an altitudinal distance of 160 m a.s.l. All others occurred at the same location.

### 3.2 Isolation by distance and resistance

**FIGURE 3:**
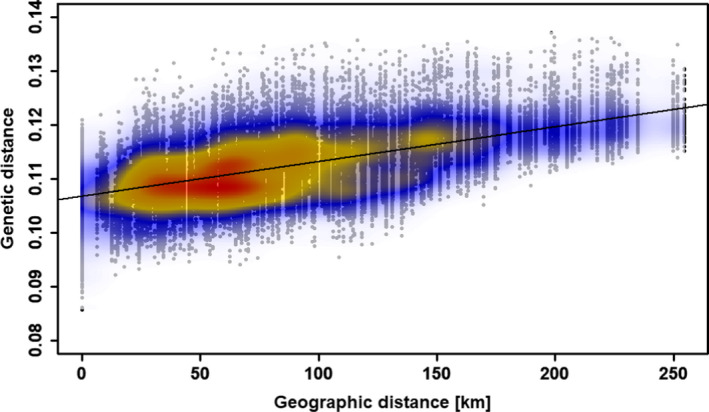
Scatterplot for isolation by geographic (Euclidean) distance. The genetic distances were calculated according to Bray–Curtis (Bray & Curtis, 1957) for pairs of individuals. Colors indicate the density of individuals; that is, red and gray illustrate the highest and lowest density of individuals, respectively

**FIGURE 4:**
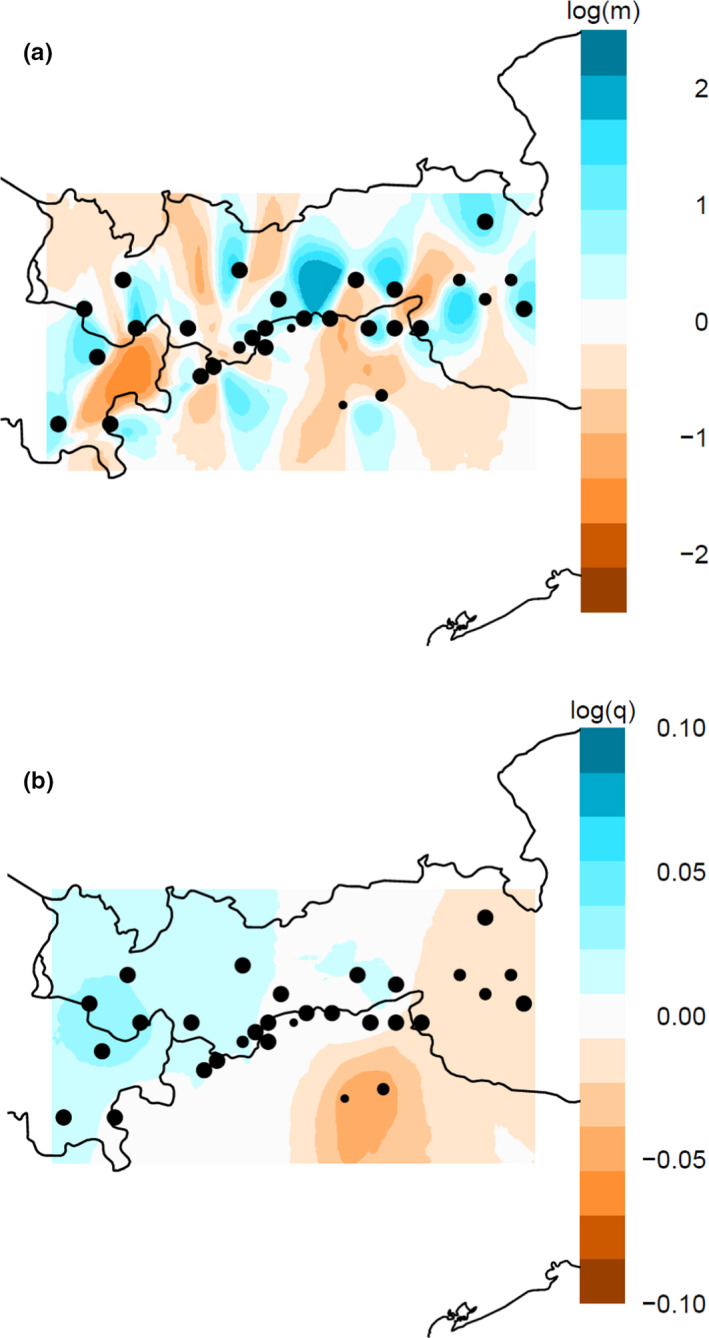
Estimated effective migration surface (eems) results based on 30 populations of *Lycaena tityrus* for the posterior mean of the effective migration surface (a) and the effective genetic diversity surface (b). In (a), the colors blue, white, and orange illustrate areas of high dispersal (dispersal corridor), isolation by distance, and low dispersal (dispersal barrier), respectively. In (b), blue and orange/brown show areas of high and low genetic diversity, respectively. The black dots indicate the population locations, and their size indicates the respective number of individuals. The black lines illustrate the national borders

Genetic distances among all individuals (Bray–Curtis) ranged from 0.086 to 0.137, and the geographic (Euclidean) distance between sampled populations ranged from 6.2 to 254.4 km. Mantel tests for isolation by distance showed a significant correlation between genetic and Euclidean distances (*r* = 0.574, *p* < .0001; Figure 3), but not between genetic and altitudinal distances (*r* = 0.033, *p* = .170). The eems analysis showed several dispersal barriers (Figure 4a). Both dolomite populations 29 and 30 were clearly separated from all others. The Inn valley and tributaries seemed to separate population 28 and populations 1–5. Additionally, populations 1–3 and 4–5 were separated by the mountains Sulzfluh (2,817 m a.s.l.), Madrisa (2,826 m a.s.l.), and Piz Buin (3,312 m a.s.l.). Other barriers in the central Alps followed mountain ridges, namely, Stubai and Ötz Alps with the Wildspitze (3,770 m a.s.l.) and the Ortler (3,905 m a.s.l.) in the south. Three migration barriers in the eastern part of our study area included the Rienz valley, separating populations 11 and 12 from the other eastern populations, the mountains Barmer Spitze (3,200 m a.s.l.) and Großvenediger (3,666 m a.s.l.), and the Großglockner (3,798 m a.s.l.) separating populations 15–16 from 17 to 18. Spatial analyses of effective genetic diversity indicated a gradient from west to east, with seven western and one central population showing a particularly high genetic diversity, while a low genetic diversity was found in the eastern‐most populations and especially in both southeastern populations (Figure 4b).

The results of the IBR analyses showed that the highest correlations for the simple Mantel tests were for altitude, slope, and tree cover (Table 3). The partial Mantel tests showed that roads and water bodies affected the genetic structure of *L. tityrus* in the European Alps (Table 3). The MLPE models for isolation by resistance, finally, showed that all environmental factors contributed to population genetic structure (Table 4).

**TABLE 3:** Results of simple and partial Mantel tests for isolation by resistance (IBR) in 30 *Lycaena tityrus* populations

Mantel		Partial Mantel			
Landscape feature	*r*	Landscape feature	Partialled out landscape feature	*r*	*p*
IBR altitude	0.5215	IBR road	IBR altitude	−0.1990	**.0001**
IBR grass (conductance)	0.3191	IBR road	IBR slope	−0.2286	**.0001**
IBR road	0.4442	IBR road	IBR tree	−0.1737	**.0001**
IBR slope	0.5375	IBR water	IBR altitude	−0.1624	**.0001**
IBR tree	0.5399	IBR water	IBR slope	−0.1613	**.0001**
IBR water	0.3697	IBR water	IBR tree	−0.2363	**.0001**

Note

Only significant partial Mantel tests are shown. Significant *p*‐values are given in bold.

**TABLE 4:** Results of maximum‐likelihood population‐effects (MLPE) models for isolation by resistance

Model	AIC	ΔAIC	*R* ^2^ *m*	*R* ^2^ *c*
GD ~ altitude + grass + road + slope + tree + water	−330,167.0	0.0	0.57773	0.90806
GD ~ altitude + grass + road + slope + water	−330,166.9	0.1	0.57750	0.90771
GD ~ altitude + grass + road + slope + tree	−329,993.6	173.4	0.56491	0.90866
GD ~ grass + road + slope + tree + water	−329,561.1	605.9	0.56816	0.90990
GD ~ altitude + grass + slope + tree + water	−327,770.8	2,396.2	0.52015	0.89926
GD ~ altitude + grass + road + tree + water	−326,885.9	3,281.1	0.50081	0.87784
GD ~ altitude + road + slope + tree + water	−325,805.6	4,361.4	0.55444	0.87370

Note

Here, we presented only the results of 6‐ and 5‐factor models. Note that model including all six factors had the lowest AIC. Models included genetic distance (GD) according to Bray–Curtis (Bray & Curtis, 1957) as response variable, altitude, grass cover, road, slope, tree cover, and/or water bodies as fixed factors, and *Lycaena tityrus* individuals (*n* = 261) as a random factor. We used the Akaike information criterion (AIC) as an indicator of model quality. The marginal *R* ^2^ (*R* ^2^ *m*) gives the proportion of variance explained by fixed factors, and the conditional *R* ^2^ (*R* ^2^ *c*) by fixed and random factors.

### 3.3 Population structure

**FIGURE 5:**
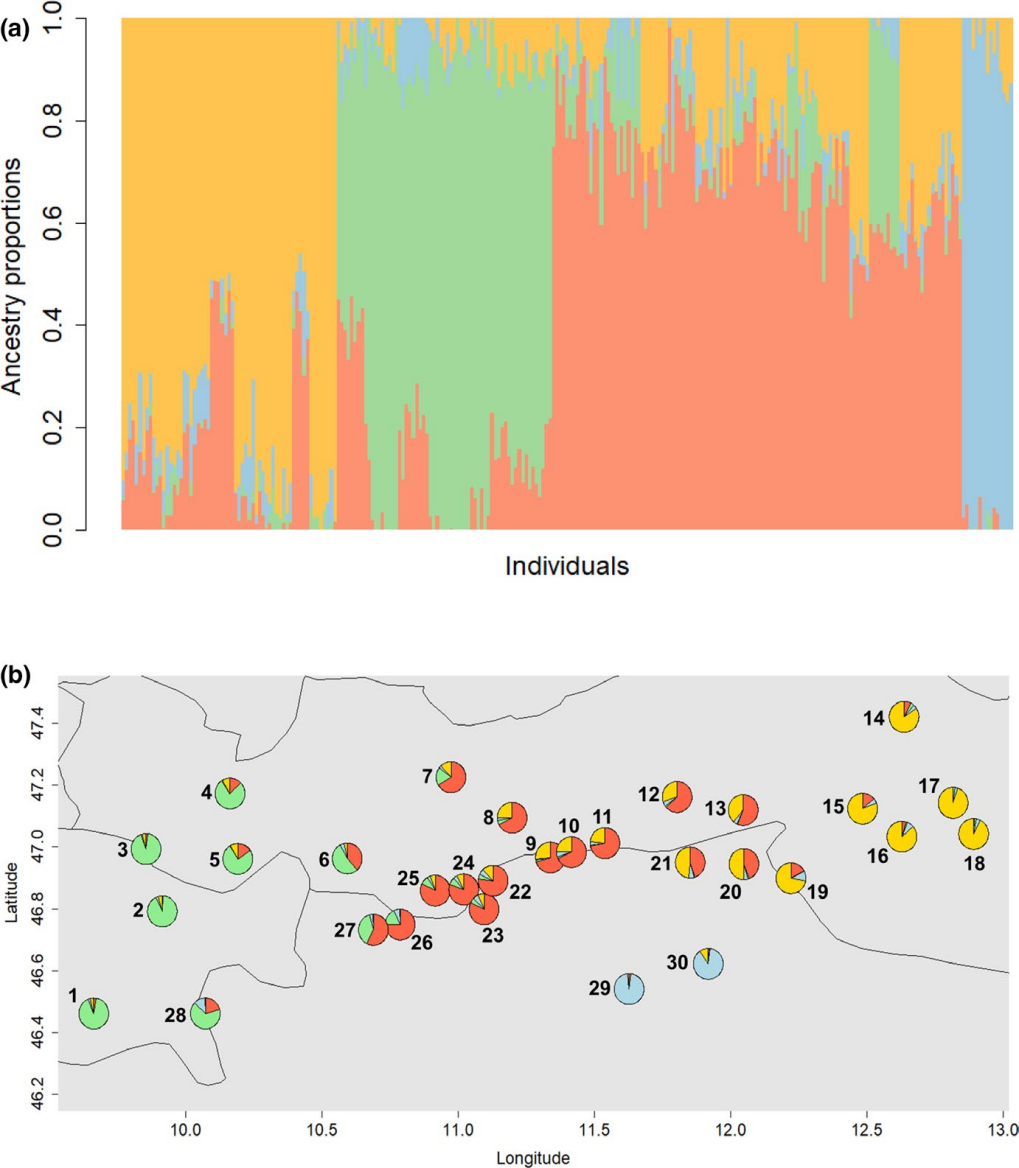
Genetic clusters as computed with a sparse non‐negative matrix factorization (*SNMF*), based on 13 455 SNPs and 30 *Lycaena tityrus* populations. Four genetic clusters were identified. In (a), each vertical line represents one individual, and its likely assignment to a specific genetic cluster encoded by different colors. In (b), each circle represents one population (cf. Figure 2), with the percentage of individuals assigned to a specific genetic cluster

**FIGURE 6:**
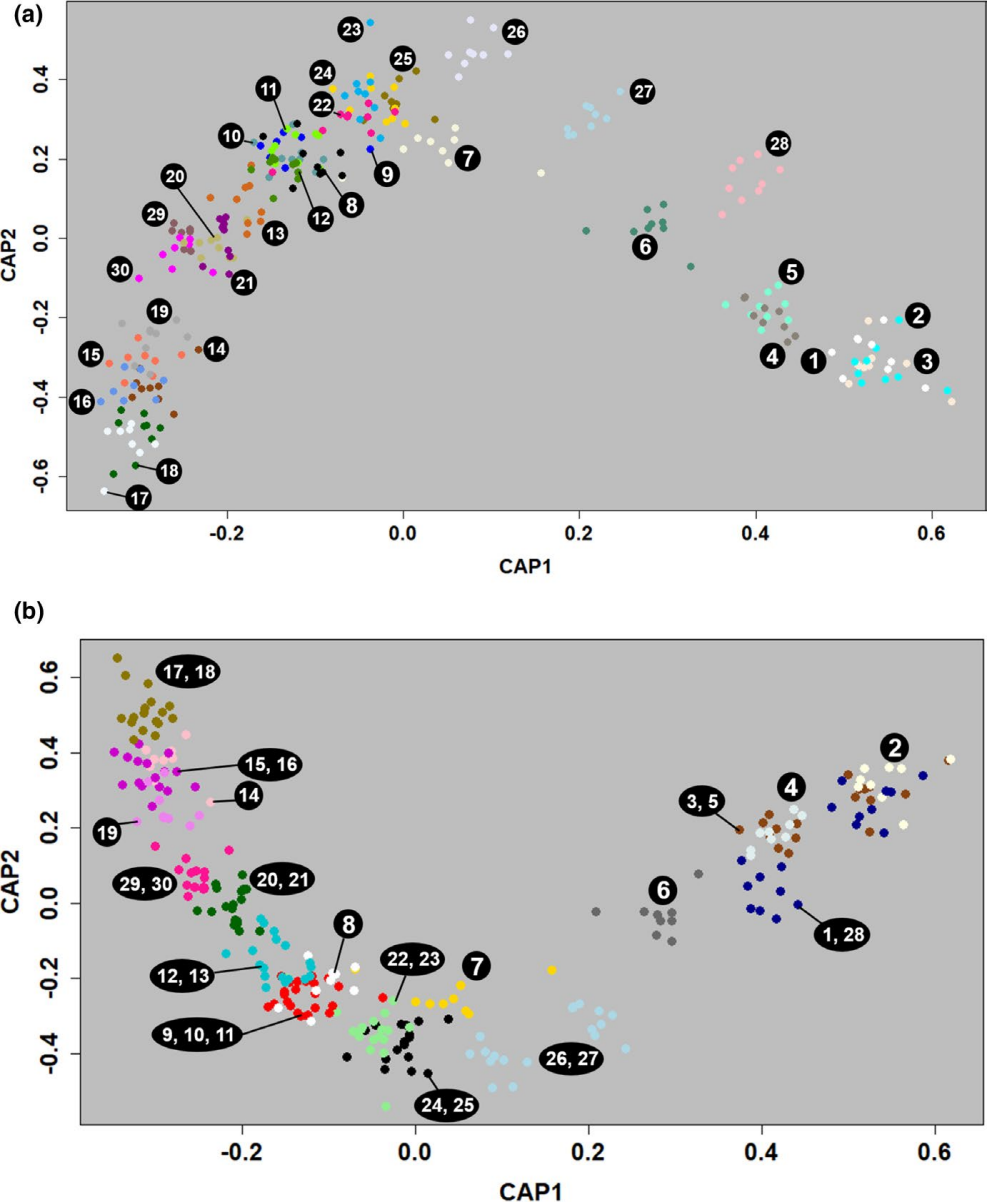
Scatterplots indicating the genetic distance of individuals across (a) the sampled locations (populations) and (b) major river valleys in combination with high mountain ridges according to distance‐based redundancy analyses. Each point represents one individual and the colors indicate (a) each population and (b) each group (across major river valleys and high mountain ranges). The canonical principal coordinate (CAP) scores are shown for the first two discriminating axes

Regarding molecular indices, populations 3 and 30 showed the highest and lowest observed heterozygosity, respectively (Table 5). The highest expected heterozygosity and inbreeding coefficient were found in population 2, while the lowest values were observed in 29. The program arlequin provided *F*_ST_ values between each population pair, and from these values, we calculated the mean *F*_ST_ value for each population. Based on arlequin and bayescan, the highest mean *F*_ST_ value was found in population 29, and the lowest one in population 13 (also 10 and 12 in bayescan; Table 5, for details see Table S1.1, Appendix S1). An AMOVA revealed that the highest amount of genetic variation occurred within individuals (94.6%), followed by a significant structuring among populations (4.5%), while variation among individuals within populations was not significant (Table 6). *SNMF* analysis resulted in four, a western (seven populations), eastern (8), southeastern (2), and a central cluster (13; Figure 5). Distance‐based redundancy analyses (dbRDA) revealed that all a priori groupings were statistically supported (Table 2). Based on effect sizes, the grouping according to sampling location is most strongly supported (Figure 6a), followed by the grouping according to river valleys in combination with high mountain ridges (Figure 6b).

**TABLE 5:** Number of individuals, observed (*H*_O_) and expected heterozygosity (*H*_E_), inbreeding coefficient (*F*_IS_), and mean *F*_ST_ calculated by arlequin (A) and bayescan (B) for 30 *Lycaena tityrus* populations

Population	Number of individuals	*H*_O_	*H*_E_	*F*_IS_	Mean *F*_ST_ (A)	Mean *F*_ST_ (B)
1	9	0.122	0.120	0.029	0.066	0.068
2	9	0.121	0.121	0.041	0.061	0.060
3	9	0.124	0.121	0.027	0.061	0.062
4	9	0.121	0.119	0.033	0.051	0.049
5	9	0.121	0.121	0.040	0.047	0.039
6	9	0.120	0.118	0.031	0.048	0.045
7	9	0.119	0.117	0.032	0.034	0.023
8	9	0.117	0.114	0.024	0.032	0.022
9	7	0.114	0.110	0.031	0.034	0.031
10	9	0.118	0.116	0.028	0.029	0.016
11	9	0.117	0.115	0.027	0.031	0.019
12	9	0.117	0.115	0.030	0.030	0.016
13	9	0.118	0.116	0.027	0.029	0.016
14	9	0.111	0.110	0.035	0.044	0.049
15	8	0.111	0.110	0.038	0.043	0.044
16	8	0.110	0.108	0.027	0.045	0.050
17	8	0.110	0.108	0.027	0.052	0.064
18	9	0.107	0.107	0.040	0.051	0.063
19	9	0.113	0.109	0.015	0.042	0.046
20	9	0.116	0.113	0.020	0.037	0.035
21	9	0.116	0.114	0.031	0.035	0.029
22	9	0.116	0.113	0.022	0.031	0.021
23	9	0.119	0.115	0.016	0.037	0.030
24	9	0.115	0.115	0.039	0.036	0.028
25	8	0.115	0.115	0.040	0.039	0.035
26	9	0.118	0.116	0.028	0.046	0.048
27	9	0.120	0.116	0.013	0.049	0.051
28	9	0.118	0.114	0.019	0.071	0.086
29	7	0.108	0.101	0.006	0.076	0.122
30	8	0.104	0.102	0.033	0.072	0.112

Note

The global *F*_ST_ among all populations is 0.04. Genetic indices were calculated with the packages “adegenet” and “hierfstat” in R.

**TABLE 6:** Results of an AMOVA for 30 populations of *Lycaena tityrus*

Source of variation	*df*	SS	Variance components	Percentage of variation	*p*
Among populations	29	40,299.4	35.7	4.49	**<.0001**
Among individuals	231	177,423.4	7.6	0.95	.1417
Within individuals	261	196,499.5	752.9	94.56	**<.0001**

Note

Given are the percentages of variation among populations, among individuals within populations, and within individuals. Significant *p*‐values are given in bold.

The DIYABC analysis showed the strongest support for scenario 5 with a posterior probability of 0.762 and confidence intervals of 0.667–0.860 (Figure S1.1, Appendix S1). Scenario 5 considered that the western, central, and eastern population clusters evolved separately, and that the southeastern population cluster split from the eastern population cluster. All other scenarios received only little support with posterior probabilities below 0.132.

### 3.4 Loci under selection according to altitude

In total, we identified 514 SNPs as potential outliers according to altitude with at least two analyses. baypass, FDIST2, and bayescan detected 673, 1,866, and 269 significant outlier SNPs, respectively. Thirty‐six out of the 514 putative outlier SNPs had a match in omicsbox (Table 7). Of these, ten could be assigned to membrane transport, four to membrane/peptidases, three to DNA binding, two each to metal binding, transcription and one each to post‐translational modification, nucleotide binding, phosphatase inhibitor, kinase activity, translation, tyrosinase, lipid binding, microtubule binding, ATP binding, protein phosphatase regulator activity, nucleosome assembly, protein dimerization activity, and protease. In *LFMM*, we detected 16 and 13 outlier SNPs being related to altitude and temperature variables, respectively (data not shown). Nine outlier SNPs were found to be linked to both altitude and different temperature variables.

**TABLE 7:** Overview of 36 outlier SNPs in *Lycaena tityrus*

SNP	Name	Definition	Main function	*F*_ST__F	*F*_ST__B	XtX
1	cce85.4	Ubiquitin‐conjugating enzyme	Post‐translational modification	0.10		34.1
2	cce4792.4	Protein Rab‐8A	Nucleotide binding	0.14		33.3
3	cce903.4	Phosphatase	Metal binding	0.12	0.10	
4	cce2716.2	Phosphatase 1 regulatory subunit	Phosphatase inhibitor	0.12		34.3
5, 6	cce3535.5	Neprilysin‐2	Membrane/peptidase	0.12, 0.23	0.12, 0.16	34.6, 33.1
7	cce6371.8	Trehalose transporter	Membrane transport	0.10		32.9
8	cce11923.10	Phosphatidylinositol 3‐kinase	Kinase activity	0.19	0.16	32.8
9	cce4686.12	Graves disease carrier protein	Membrane transport	0.19	0.13	
10, 11	cce3911.10	Inositol 1,4,5‐trisphosphate receptor	Membrane transport	0.10, 0.14	0.13	37.6
12	cce19073.2	Arginine/serine‐rich protein PNISR	Translation	0.22	0.14	34.6
13	cce3081.1	Phenoloxidase subunit 1	Tyrosinase	0.17	0.15	34.8
14	cce302662.1	Synaptotagmin‐10	Membrane/peptidase	0.18	0.16	34.6
15	cce269.7	Zinc‐finger protein 674	Transcription	0.20	0.18	
16	cce5955.2	SEC14‐like protein 2	Lipid binding	0.18		33.1
17	cce1202.2	ATP‐binding cassette	Membrane transport	0.12		33.1
18	cce73.16	Putative tyrosine‐protein kinase	Membrane transport	0.14	0.11	33.5
19	cce11385.6	Glycoprotein‐N‐acetylgalactosamine 3‐beta‐galactosyltransferase 1	Membrane/peptidase	0.13	0.13	
20	cce1920.4	Microtubule‐associated protein	Actin, kinase, microtubule & tubulin binding	0.10	0.10	
21	cce3888.3	Monocarboxylate transporter	Membrane transport	0.14		33.1
22	cce24096.1	ATP‐binding cassette subfamily A member 3‐like	Membrane transport	0.18		34.6
23	cce8343.10	Ubiquitin‐conjugating enzyme	ATP binding	0.11		34.1
24	cce2777.3	Ubiquitin‐protein ligase	Metal binding	0.13	0.11	
25	cce1435.8	Potassium voltage‐gated channel	Membrane transport	0.17	0.14	
26	cce2484.3	Serine/threonine protein phosphatase	Protein phosphatase regulator activity	0.12	0.13	
27	cce2118.6	Longitudinal lacking protein	DNA binding	0.21		39.0
28	cce515.22	Nucleosome assembly protein	Nucleosome assembly	0.13		33.6
29	cce6093.5	Desert hedgehog protein B	Membrane/peptidase	0.15		32.9
30	cce181953.4	Longitudinal lacking protein	DNA binding	0.10		32.7
31	cce10.2	Protein abrupt	DNA binding	0.11	0.11	
32	cce1246.8	Transcription factor AP‐4	Protein dimerization activity	0.16	0.14	32.9
33	cce302735.7	Bark beetle isoform X2	Membrane transport	0.20		33.0
34	cce1678.5	Negative elongation factor A	Transcription	0.15	0.11	
35	cce1712.1	Cysteine proteinase inhibitor	Protease	0.10		33.2
36	cce12716.1	Sodium leak channel	Membrane transport	0.20	0.16	33.7

Note

All SNPs mentioned here were verified with at least two out of three *F*_ST_ outlier tests (baypass, FDIST2, bayescan), had an *F*_ST_ value > 0.1, and their gene orthologues were obtained with omicsbox version 1.3 (for more details see Table S1.2, Appendix S1). This analysis is based on the putative *Calycopis cecrops* (Lepidoptera: Lycaenidae) v. 1.1 genome annotation. The rank of the outlier SNPs is indicated by their *F*_ST_ values calculated by FDIST2 (*F*_ST__F) and bayescan (*F*_ST__B), and XtX values calculated by baypass. The higher the *F*_ST_ or XtX value, the higher the importance of the outlier SNP.

## 4 DISCUSSION

Our results show a pronounced genetic structure of *L. t. subalpinus* populations in the European Alps, indicated by (a) relatively high *F*_ST_ values between populations, (b) evidence for isolation by distance and resistance, (c) genetic barriers separating populations and population clusters (dbRDA and eems analysis), and (d) a high number of half‐siblings within populations. These findings are expected if *L. t. subalpinus* is a relatively sedentary butterfly forming closed populations (Ricketts, 2001; Trense et al., 2021). Nevertheless, we found four putative half‐sibling pairs with both individuals being located in different populations, in all cases involving one individual in population 29 and one in population 30, suggesting gene flow over a distance of 24 km. This suggests occasional long‐range dispersal despite the species mainly being sedentary. The two populations are separated from all others by a large river valley, have low levels of heterozygosity, show comparatively high mean *F*_ST_ values when compared to the other populations, and form a distinct genetic cluster (Table 5, Figure 5).

Overall, landscape features (altitude, forests, slope, roads, and water bodies) seemed to have a large impact on the genetic structure of *L. tityrus*. Similarly, altitude, forest, and roads have been found to influence the genetic structure of *L. tityrus* within one Alpine valley in a previous study (Trense et al., 2021). Note here that the results of simple and partial Mantel tests need to be interpreted with caution because they suffer from high Type I error rates (Cushman et al., 2013; Guillot & Rousset, 2013). However, the MLPE models indicated that all factors contributed to the population genetic structure of *L. tityrus* in the European Alps. In particular, high mountains and large river valleys seem to represent important barriers hampering gene flow, which is supported by our dbRDA and eems results (Table 2, Figures 4 and 6). High mountains above ca. 2,300–2,500 m a. s. l. do not comprise suitable habitats for *L. t. subalpinus*, especially when covered by glaciers. Accordingly, mountain ranges may act as effective barriers for dispersal and thus gene flow among bee, butterfly, and grasshopper populations at large scales (Britten et al., 1995; Després et al., 2019; Hewitt, 1996; Jaffé et al., 2019). Our study shows that this applies also to smaller spatial scales within mountain ranges. Likewise, large rivers such as Danube, Isère, Rhine, and Rhone are known to affect large‐scale genetic patterns, indicated by genetic differentiation of insect populations from either side of rivers (Cupedo & Doorenweerd, 2020; Mardulyn, 2001; Schmitt et al., 2007). However, smaller rivers were not found to comprise a barrier for *L. t. subalpinus* in one Austrian valley (Trense et al., 2021). Hence, whether a river valley displays a barrier to gene flow depends on its size, elevation, and climatic conditions (Ćosić et al., 2013; Link et al., 2015; Muñoz‐Mendoza et al., 2017). In *L. t. subalpinus*, only large river valleys at low altitudes seem to act as barriers. These currently do not offer suitable habitat for the species, mainly due to agricultural intensification, although they may have historically.

Cluster analyses revealed a western, eastern, southeastern, and a central cluster, which may indicate different refuges from which the Alps were recolonized independently after the last glacial period. A similar pattern was found in *Erebia* butterflies (Schmitt et al., 2016), and an eastern and western genetic cluster occurred also in alpine *Erebia alberganus* (Louy et al., 2014) and in *Drusus discolor* (Pauls et al., 2006). Accordingly, the Diyabc analysis suggested the occurrence of three glacial refuges in the western, central (perhaps along the river Etsch), and eastern Alps, with the southeastern population cluster originating from the eastern refuge. Interestingly, the different genetic clusters in our study are characterized by striking differences in genetic diversity, being high in the western, intermediate in the central, and low in the eastern and especially in the southeastern cluster. These data suggest that the largest glacial refuge of *L. t. subalpinus* was located in southwestern Europe or at the southwestern edge of the Alps. This refuge may have included the lowlands between the western Alps and the Pyrenees, as the alpine subspecies occurs in both mountain areas, which may thus indicate relict populations (Tolman & Lewington, 2008). In addition, admixture of different western (and southwestern) refuges or hybridization with low‐altitude populations, that is, *L. tityrus tityrus*, may have contributed to the high genetic diversity in the western Alps. Generally, the southwestern Alps are known to be important evolutionary centers for arctic–alpine species (Louy et al., 2014; Schmitt, 2009; Schönswetter et al., 2005). The lower genetic diversity in the central and especially eastern genetic clusters suggests a smaller refuge size (or rather smaller effective population sizes) as compared with the supposed western refuge, in combination with founder effects for the southeastern cluster (Austerlitz et al., 1997; Fayard et al., 2009). The according dolomite populations may thus have been founded by relatively few individuals from the eastern population cluster and host plants are also rarer in the eastern part of the European Alps, explaining their low genetic diversity and high similarity of both respective populations but strong differentiation from all others. This would be consistent with the general trend of a decrease in genetic diversity from the core to the edges of a species’ range (Brown et al., 1995; Eckert et al., 2008). We can also not rule out that our eastern sampling points were further away from the eastern refuge, for instance located in the Slovenian Alps, explaining the decreased genetic diversity.

In addition to effects of the last glaciation, the patterns found could be also influenced by more recent developments. In the dolomites, for instance, *L. t. subalpinus* is much rarer than further north, which is most likely due to differences in geology. The dolomites with their karst landscapes have much dryer soils strongly reducing the abundance of *Rumex* plants compared with the central Alps with their crystalline bedrocks. Concomitantly, population size and connectivity are likely to be low in the dolomites, which may also explain the low genetic diversity of these populations. Whether this may also apply to (parts of) the eastern cluster is currently unclear. Thus, while the large‐scale population structure of *L. t. subalpinus* is likely shaped by the last glacial period, local and regional factors may also be important.

With regard to the conservation status of the taxon, *L. t. subalpinus* represents an evolutionary significant unit for conservation, based on substantial morphological, ecological, and genetic differences compared to the lowland form *L. t. tityrus* (Karl et al., 2008, 2009; Tolman & Lewington, 2008). Whether this also applies to the inner‐Alpine genetic clusters found here is open to debate. We suggest that the dolomite populations should be considered as their own entities, as these show strong genetic divergence in the *SNMF* analysis and are likely more threatened than the central Alpine populations. For the protection of the taxon *L. t. subalpinus* though, conservation efforts should be concentrated in the western Alps with their genetically diverse populations and the importance of genetic diversity in future adaptation.

As predicted, we found footprints of selection to the unique alpine environments including differences in temperature, oxygen, ultraviolet radiation, and food availability (Cheviron & Brumfield, 2012; Dillon et al., 2006; Montero‐Mendieta et al., 2019). Overall, 514 outlier loci out of 13 455 SNPs were linked to altitudinal differences. Since no annotated genome is available for *L. tityrus*, we found putative functions in only 36 outlier loci, 14 out of which were associated with membrane‐related proteins or functions. Similarly, five out of 11 outlier loci with putative functions were found to be membrane‐related proteins in a previous study (Trense et al., 2021). Nevertheless, no common outlier SNPs were found in both the current and the previous study. The missing overlap of outlier SNPs may result from selection and other processes differing between the scales investigated, leading to different outliers being detected. In the previous study, we sampled two contiguous subvalleys, with accordingly little overall genetic divergence such that outliers may represent more recent selection events (Trense et al., 2021). In the present study, we sampled several different sites in the European Alps, which are genetically clearly differentiated, thus representing a longer history of genetic differentiation. It is also likely that the outlier loci detected in the current study are associated with environmental variables that vary substantially at a broader scale. This may include selection on membrane features along the altitudinal gradient, probably in relation to membrane fluidity, which affects cold tolerance in ectotherms (Hazel, 1995; Hochachka & Somero, 2002). Higher proportions of unsaturated fatty acids in the membrane increase the membrane fluidity and thus cold tolerance (Brown et al., 2019; Haubert et al., 2008; Ohtsu et al., 1998). We were also able to show here that some outlier SNPs linked to altitude were also linked to temperature, suggesting thermal selection. Interestingly, five other outlier loci, the serine/threonine protein and phosphatidylinositol phosphatase/kinase, the zinc‐finger protein, and other potassium channels and phenoloxidases were also detected in other studies on insects as potential outlier loci in relation to altitude (Jackson et al., 2020; Montero‐Mendieta et al., 2019; Trense et al., 2021; Waldvogel et al., 2018). These outlier loci are involved in several cellular processes (Cassandri et al., 2017; MacKinnon, 2003; Wera & Hemmings, 1995).

## 5 CONCLUSIONS

This study shows high genetic differentiation between *L. t. subalpinus* populations in the European Alps. Large‐scale patterns were likely shaped by the last glacial period and the location of refuges in the western, central, and eastern Alps. At a smaller scale, high mountain ridges and large river valleys limit gene flow and thus structure populations. We suggest that conservation efforts should focus on the western Alps based on the high genetic diversity found there. Furthermore, the dolomite populations could be treated as separate management unit. Our findings demonstrate the usefulness of genome‐wide SNPs for estimating population structure, constraints on dispersal, and selection in the wild. In times of global climate change, it is important to better understand the population genetic structure of alpine species, because they seem to be particularly vulnerable to global warming (Engler et al., 2011; Hoffmann, 2010; Schmitt et al., 2014).

## CONFLICT OF INTEREST

None declared.

## AUTHOR CONTRIBUTIONS

**Daronja Trense:** Conceptualization (equal); Data curation (lead); Formal analysis (lead); Investigation (lead); Methodology (lead); Visualization (lead); Writing‐original draft (equal); Writing‐review & editing (equal). **Ary A. Hoffmann:** Conceptualization (equal); Data curation (equal); Formal analysis (equal); Software (equal); Visualization (equal); Writing‐review & editing (equal). **Klaus Fischer:** Conceptualization (equal); Data curation (equal); Formal analysis (equal); Funding acquisition (lead); Investigation (equal); Methodology (equal); Project administration (lead); Software (equal); Visualization (equal); Writing‐original draft (equal); Writing‐review & editing (equal).

## Supporting information

Appendix S1

## Data Availability

Data from this study are available from the Dryad Digital Repository: https://doi.org/10.5061/dryad.4b8gthtdh.
