# Supplementary material for: Large‐ and small‐scale geographic structures affecting genetic patterns across populations of an Alpine butterfly
Source: Ecol Evol. 2021 Sep 28;11(21):14697–714. doi: 10.1002/ece3.8157 (PMC8571576; doi:10.1002/ece3.8157)
Supplement: Supplementary file 1 — Appendix S1 [file ECE3-11-14697-s001.docx]

**Large- and small-scale geographic structures affect genetic patterns across populations of an Alpine butterfly**

**FIGURE S1.1** Selected scenarios of population demographic history based on DIYABC analyses. Seven tested scenarios for four population clusters in *Lycaena tityrus* were considered. t1 and t2 reflect the time scale measured in generations. N1, N2, N3, N4, and NA are the effective population sizes of the corresponding populations in the west (Pop 1), central north (Pop 2), east (Pop 3), southeast (Pop 4), and of the ancestral population.


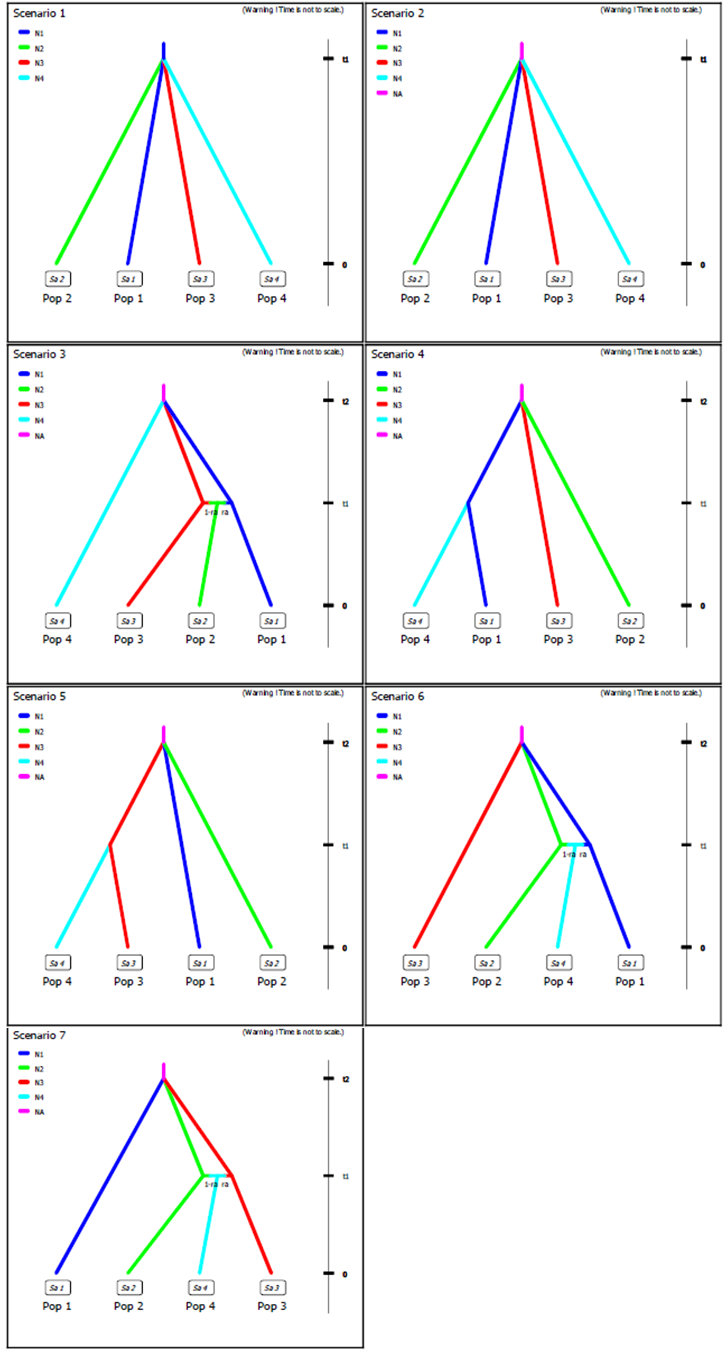


**TABLE S1.1** Genetic differentiation among 30 *Lycaena tityrus* populations based on 13 455 SNPs. Pairwise *F_ST_* values are shown below the diagonal. Significant *F_ST_*-values after Bonferroni-Holm correction are given in bold

|  | **1** | **2** | **3** | **4** | **5** | **6** | **7** | **8** | **9** | **10** | **11** | **12** | **13** | **14** | **15** | **16** | **17** | **18** | **19** | **20** | **21** | **22** | **23** | **24** | **25** | **26** | **27** | **28** | **29** | **30** |
| --- | --- | --- | --- | --- | --- | --- | --- | --- | --- | --- | --- | --- | --- | --- | --- | --- | --- | --- | --- | --- | --- | --- | --- | --- | --- | --- | --- | --- | --- | --- |
| **1** | 0 |  |  |  |  |  |  |  |  |  |  |  |  |  |  |  |  |  |  |  |  |  |  |  |  |  |  |  |  |  |
| **2** | **0.0120** | 0 |  |  |  |  |  |  |  |  |  |  |  |  |  |  |  |  |  |  |  |  |  |  |  |  |  |  |  |  |
| **3** | **0.0163** | **0.0054** | 0 |  |  |  |  |  |  |  |  |  |  |  |  |  |  |  |  |  |  |  |  |  |  |  |  |  |  |  |
| **4** | **0.0263** | **0.0141** | **0.0129** | 0 |  |  |  |  |  |  |  |  |  |  |  |  |  |  |  |  |  |  |  |  |  |  |  |  |  |  |
| **5** | **0.0212** | **0.0092** | **0.0090** | **0.0062** | 0 |  |  |  |  |  |  |  |  |  |  |  |  |  |  |  |  |  |  |  |  |  |  |  |  |  |
| **6** | **0.0405** | **0.0320** | **0.0331** | **0.0232** | **0.0210** | 0 |  |  |  |  |  |  |  |  |  |  |  |  |  |  |  |  |  |  |  |  |  |  |  |  |
| **7** | **0.0503** | **0.0474** | **0.0460** | **0.0347** | **0.0298** | **0.0312** | 0 |  |  |  |  |  |  |  |  |  |  |  |  |  |  |  |  |  |  |  |  |  |  |  |
| **8** | **0.0641** | **0.0608** | **0.0600** | **0.0475** | **0.0409** | **0.0402** | **0.0172** | 0 |  |  |  |  |  |  |  |  |  |  |  |  |  |  |  |  |  |  |  |  |  |  |
| **9** | **0.0673** | **0.0648** | **0.0637** | **0.0494** | **0.0450** | **0.0417** | **0.0211** | **0.0137** | 0 |  |  |  |  |  |  |  |  |  |  |  |  |  |  |  |  |  |  |  |  |  |
| **10** | **0.0639** | **0.0601** | **0.0594** | **0.0463** | **0.0417** | **0.0392** | **0.0146** | **0.0091** | **0.0060** | 0 |  |  |  |  |  |  |  |  |  |  |  |  |  |  |  |  |  |  |  |  |
| **11** | **0.0661** | **0.0623** | **0.0607** | **0.0470** | **0.0435** | **0.0400** | **0.0185** | **0.0060** | **0.0094** | 0.0034 | 0 |  |  |  |  |  |  |  |  |  |  |  |  |  |  |  |  |  |  |  |
| **12** | **0.0664** | **0.0610** | **0.0620** | **0.0479** | **0.0427** | **0.0400** | **0.0163** | **0.0097** | **0.0080** | 0.0039 | 0.0036 | 0 |  |  |  |  |  |  |  |  |  |  |  |  |  |  |  |  |  |  |
| **13** | **0.0692** | **0.0629** | **0.0621** | **0.0494** | **0.0439** | **0.0417** | **0.0163** | **0.0078** | **0.0085** | 0.0022 | **0.0072** | 0.0026 | 0 |  |  |  |  |  |  |  |  |  |  |  |  |  |  |  |  |  |
| **14** | **0.0892** | **0.0848** | **0.0848** | **0.0709** | **0.0650** | **0.0629** | **0.0387** | **0.0312** | **0.0307** | **0.0254** | **0.0266** | **0.0249** | **0.0186** | 0 |  |  |  |  |  |  |  |  |  |  |  |  |  |  |  |  |
| **15** | **0.0887** | **0.0857** | **0.0843** | **0.0692** | **0.0662** | **0.0623** | **0.0398** | **0.0265** | **0.0290** | **0.0232** | **0.0273** | **0.0222** | **0.0164** | **0.0174** | 0 |  |  |  |  |  |  |  |  |  |  |  |  |  |  |  |
| **16** | **0.0923** | **0.0886** | **0.0883** | **0.0760** | **0.0708** | **0.0657** | **0.0440** | **0.0306** | **0.0328** | **0.0259** | **0.0299** | **0.0270** | **0.0199** | **0.0141** | **0.0061** | 0 |  |  |  |  |  |  |  |  |  |  |  |  |  |  |
| **17** | **0.0965** | **0.0934** | **0.0916** | **0.0784** | **0.0730** | **0.0706** | **0.0498** | **0.0387** | **0.0405** | **0.0346** | **0.0373** | **0.0333** | **0.0279** | **0.0142** | **0.0203** | **0.0172** | 0 |  |  |  |  |  |  |  |  |  |  |  |  |  |
| **18** | **0.0963** | **0.0915** | **0.0926** | **0.0787** | **0.0736** | **0.0723** | **0.0484** | **0.0375** | **0.0404** | **0.0335** | **0.0353** | **0.0324** | **0.0287** | **0.0180** | **0.0191** | **0.0184** | **0.0093** | 0 |  |  |  |  |  |  |  |  |  |  |  |  |
| **19** | **0.0876** | **0.0832** | **0.0847** | **0.0687** | **0.0670** | **0.0613** | **0.0381** | **0.0275** | **0.0271** | **0.0208** | **0.0255** | **0.0201** | **0.0149** | **0.0190** | **0.0113** | **0.0121** | **0.0240** | **0.0223** | 0 |  |  |  |  |  |  |  |  |  |  |  |
| **20** | **0.0771** | **0.0739** | **0.0733** | **0.0586** | **0.0552** | **0.0516** | **0.0306** | **0.0209** | **0.0224** | **0.0137** | **0.0189** | **0.0141** | **0.0073** | **0.0269** | **0.0211** | **0.0231** | **0.0331** | **0.0326** | **0.0166** | 0 |  |  |  |  |  |  |  |  |  |  |
| **21** | **0.0748** | **0.0697** | **0.0702** | **0.0568** | **0.0514** | **0.0469** | **0.0260** | **0.0169** | **0.0178** | **0.0121** | **0.0169** | **0.0126** | **0.0066** | **0.0224** | **0.0181** | **0.0203** | **0.0309** | **0.0301** | **0.0178** | **0.0066** | 0 |  |  |  |  |  |  |  |  |  |
| **22** | **0.0598** | **0.0565** | **0.0552** | **0.0436** | **0.0395** | **0.0387** | **0.0157** | **0.0142** | **0.0129** | **0.0074** | **0.0110** | **0.0119** | **0.0095** | **0.0339** | **0.0289** | **0.0335** | **0.0417** | **0.0413** | **0.0281** | **0.0208** | **0.0168** | 0 |  |  |  |  |  |  |  |  |
| **23** | **0.0653** | **0.0590** | **0.0603** | **0.0482** | **0.0439** | **0.0430** | **0.0227** | **0.0192** | **0.0191** | **0.0169** | **0.0187** | **0.0183** | **0.0175** | **0.0408** | **0.0367** | **0.0431** | **0.0519** | **0.0494** | **0.0362** | **0.0281** | **0.0235** | **0.0078** | 0 |  |  |  |  |  |  |  |
| **24** | **0.0638** | **0.0574** | **0.0584** | **0.0447** | **0.0395** | **0.0389** | **0.0162** | **0.0167** | **0.0171** | **0.0168** | **0.0178** | **0.0170** | **0.0179** | **0.0406** | **0.0374** | **0.0417** | **0.0470** | **0.0492** | **0.0372** | **0.0292** | **0.0268** | **0.0132** | **0.0198** | 0 |  |  |  |  |  |  |
| **25** | **0.0624** | **0.0559** | **0.0571** | **0.0451** | **0.0412** | **0.0429** | **0.0189** | **0.0234** | **0.0253** | **0.0200** | **0.0219** | **0.0241** | **0.0214** | **0.0441** | **0.0426** | **0.0457** | **0.0545** | **0.0542** | **0.0415** | **0.0328** | **0.0299** | **0.0192** | **0.0235** | **0.0089** | 0 |  |  |  |  |  |
| **26** | **0.0597** | **0.0579** | **0.0593** | **0.0484** | **0.0428** | **0.0420** | **0.0310** | **0.0343** | **0.0330** | **0.0307** | **0.0309** | **0.0311** | **0.0351** | **0.0575** | **0.0564** | **0.0590** | **0.0666** | **0.0679** | **0.0543** | **0.0449** | **0.0406** | **0.0276** | **0.0288** | **0.0310** | **0.0314** | 0 |  |  |  |  |
| **27** | **0.0522** | **0.0464** | **0.0447** | **0.0388** | **0.0330** | **0.0302** | **0.0333** | **0.0386** | **0.0387** | **0.0376** | **0.0389** | **0.0370** | **0.0424** | **0.0634** | **0.0617** | **0.0651** | **0.0736** | **0.0705** | **0.0621** | **0.0496** | **0.0491** | **0.0336** | **0.0388** | **0.0381** | **0.0387** | **0.0329** | 0 |  |  |  |
| **28** | **0.0426** | **0.0457** | **0.0481** | **0.0524** | **0.0448** | **0.0527** | **0.0577** | **0.0652** | **0.0711** | **0.0632** | **0.0672** | **0.0661** | **0.0705** | **0.0929** | **0.0903** | **0.0943** | **0.1018** | **0.1009** | **0.0910** | **0.0800** | **0.0775** | **0.0593** | **0.0620** | **0.0642** | **0.0666** | **0.0538** | **0.0487** | 0 |  |  |
| **29** | **0.1161** | **0.1136** | **0.1126** | **0.1009** | **0.0974** | **0.0913** | **0.0720** | **0.0642** | 0.0687 | **0.0579** | **0.0604** | **0.0591** | **0.0556** | **0.0661** | **0.0663** | **0.0668** | **0.0752** | **0.0753** | **0.0605** | **0.0637** | **0.0583** | **0.0619** | **0.0679** | **0.0701** | **0.0723** | **0.0801** | **0.0849** | **0.1124** | 0 |  |
| **30** | **0.1156** | **0.1108** | **0.1108** | **0.0981** | **0.0941** | **0.0895** | **0.0674** | **0.0578** | **0.0659** | **0.0548** | **0.0569** | **0.0540** | **0.0524** | **0.0619** | **0.0602** | **0.0598** | **0.0696** | **0.0707** | **0.0554** | **0.0609** | **0.0548** | **0.0579** | **0.0639** | **0.0669** | **0.0727** | **0.0784** | **0.0862** | **0.1094** | **0.0380** | 0 |

**TABLE S1.2** Gene information for 36 adaptive SNPs with F_ST_ > 0.1 in *Lycaena tityrus*. All SNPs mentioned here were verified with at least two different *F_ST_* outlier tests (Baypass, FDIST2, Bayescan). Gene names and gene orthologues were obtained with Omicsbox version 1.3 and are based on the putative *Calycopis cecrops* (Lepidoptera: Lycaenidae) v. 1.1 genome annotation

| **SNP** | **Name** | **Definition** | **GO IDs** | **GO Description** | **Enzyme names** |
| --- | --- | --- | --- | --- | --- |
| 1 | cce85.4 | ubiquitin-conjugating enzyme E2 variant 2 |  |  |  |
| 2 | cce4792.4 | ras-related protein Rab-8A isoform X2 | GO:0003924; GO:0005525 | GTPase activity; GTP binding | Nucleoside-triphosphate phosphatase |
| 3 | cce903.4 | protein phosphatase 1E-like | GO:0006470; GO:0004722; GO:0046872 | protein dephosphorylation; protein serine/threonine phosphatase activity; metal ion binding | Protein-serine/threonine phosphatase |
| 4 | cce2716.2 | protein phosphatase 1 regulatory subunit 12B isoform X7 |  |  |  |
| 5, 6 | cce3535.5 | neprilysin-2 isoform X1 | GO:0006508; GO:0004222; GO:0016021 | proteolysis; metalloendopeptidase activity; integral component of membrane | Peptidases |
| 7 | cce6371.8 | facilitated trehalose transporter Tret1-like | GO:0055085; GO:0022857; GO:0016021 | transmembrane transport; transmembrane transporter activity; integral component of membrane |  |

| **SNP** | **Name** | **Definition** | **GO IDs** | **GO Description** | **Enzyme names** |
| --- | --- | --- | --- | --- | --- |
| 8 | cce11923.10 | phosphatidylinositol 3-kinase catalytic subunit type 3 | GO:0036092; GO:0046854; GO:0048015; GO:0005524; GO:0016303 | phosphatidylinositol-3-phosphate biosynthetic process; phosphatidylinositol phosphorylation; phosphatidylinositol-mediated signaling; ATP binding; 1-phosphatidylinositol-3-kinase activity | Phosphatidylinositol 3-kinase |
| 9 | cce4686.12 | graves disease carrier protein-like isoform X1 | GO:0015867; GO:0055085; GO:0005347; GO:0005743; GO:0016021 | ATP transport; transmembrane transport; ATP transmembrane transporter activity; mitochondrial inner membrane; integral component of membrane |  |

**TABLE S1.2** continued

**TABLE S1.2** continued

| **SNP** | **Name** | **Definition** | **GO IDs** | **GO Description** | **Enzyme names** |
| --- | --- | --- | --- | --- | --- |
| 10, 11 | cce3911.10 | inositol 1,4,5-trisphosphate receptor isoform X1 | GO:0048016; GO:0070588; GO:0005220; GO:0070679; GO:0005783; GO:0016021 | inositol phosphate-mediated signaling; calcium ion transmembrane transport; inositol 1,4,5-trisphosphate-sensitive calcium-release channel activity; inositol 1,4,5 trisphosphate binding; endoplasmic reticulum; integral component of membrane |  |
| 12 | cce19073.2 | arginine/serine-rich protein PNISR | GO:0006412; GO:0003735; GO:0005840 | translation; structural constituent of ribosome; ribosome |  |
| 13 | cce3081.1 | phenoloxidase subunit 1 | GO:0006583; GO:0006952; GO:0055114; GO:0004503; GO:0046872; GO:0005576 | melanin biosynthetic process from tyrosine; defense response; oxidation-reduction process; monophenol monooxygenase activity; metal ion binding; extracellular region | Tyrosinase |
| 14 | cce302662.1 | synaptotagmin-10-like isoform X1 | GO:0007340; GO:0016021 | acrosome reaction; integral component of membrane |  |
| 15 | cce269.7 | zinc finger protein 674-like |  |  |  |
| 16 | cce5955.2 | SEC14-like protein 2 |  |  |  |

**TABLE S1.2** continued

| **SNP** | **Name** | **Definition** | **GO IDs** | **GO Description** | **Enzyme names** |
| --- | --- | --- | --- | --- | --- |
| 17 | cce1202.2 | ATP-binding cassette sub-family A member 1-like | GO:0055085; GO:0000166; GO:0005524; GO:0016887; GO:0042626; GO:0016020; GO:0016021 | transmembrane transport; nucleotide binding; ATP binding; ATPase activity; ATPase-coupled transmembrane transporter activity; membrane; integral component of membrane |  |
| 18 | cce73.16 | putative tyrosine-protein kinase Wsck | GO:0006468; GO:0004672; GO:0005524; GO:0016021 | protein phosphorylation; protein kinase activity; ATP binding; integral component of membrane |  |
| 19 | cce11385.6 | glycoprotein-N-acetylgalactosamine 3-beta-galactosyltransferase 1-like isoform X3 | GO:0016266; GO:0016263; GO:0016020 | O-glycan processing; glycoprotein-N-acetylgalactosamine 3-beta-galactosyltransferase activity; membrane | N-acetylgalactosaminide beta-1,3-galactosyltransferase |
| 20 | cce1920.4 | microtubule-associated protein futsch isoform X4 |  |  |  |
| 21 | cce3888.3 | monocarboxylate transporter 10-like isoform X2 | GO:0055085; GO:0022857; GO:0016021 | transmembrane transport; transmembrane transporter activity; integral component of membrane |  |

| **SNP** | **Name** | **Definition** | **GO IDs** | **GO Description** | **Enzyme names** |
| --- | --- | --- | --- | --- | --- |
| 22 | cce24096.1 | ATP-binding cassette sub-family A member 3-like | GO:0055085; GO:0005524; GO:0016887; GO:0042626; GO:0016021 | transmembrane transport; ATP binding; ATPase activity; ATPase-coupled transmembrane transporter activity; integral component of membrane | Adenosinetriphosphatase; Nucleoside-triphosphate phosphatase |
| 23 | cce8343.10 | ubiquitin-conjugating enzyme E2 R2 | GO:0005524; GO:0016740 | ATP binding; transferase activity |  |
| 24 | cce2777.3 | E3 ubiquitin-protein ligase MYCBP2 isoform X7 |  |  |  |
| 25 | cce1435.8 | potassium voltage-gated channel subfamily KQT member 1 isoform X4 | GO:0071805; GO:0005249; GO:0008076 | potassium ion transmembrane transport; voltage-gated potassium channel activity; voltage-gated potassium channel complex |  |
| 26 | cce2484.3 | serine/threonine-protein phosphatase PP2A 65 kDa regulatory subunit |  |  |  |
| 27 | cce2118.6 | longitudinals lacking protein, isoforms H/M/V-like isoform X2 |  |  |  |
| 28 | cce515.22 | nucleosome assembly protein 1-like 1 isoform X1 | GO:0006334; GO:0005634 | nucleosome assembly; nucleus |  |

**TABLE S1.2** continued

| **SNP** | **Name** | **Definition** | **GO IDs** | **GO Description** | **Enzyme names** |
| --- | --- | --- | --- | --- | --- |
| 29 | cce6093.5 | desert hedgehog protein B | GO:0007267; GO:0007275; GO:0016539; GO:0016540; GO:0008233; GO:0005615; GO:0005886 | cell-cell signaling; multicellular organism development; intein-mediated protein splicing; protein autoprocessing; peptidase activity; extracellular space; plasma membrane |  |
| 30 | cce181953.4 | longitudinals lacking protein-like isoform X1 |  |  |  |
| 31 | cce10.2 | protein abrupt-like isoform X1 |  |  |  |
| 32 | cce1246.8 | Transcription factor AP-4 | GO:0046983 | protein dimerization activity |  |
| 33 | cce302735.7 | protein bark beetle isoform X2 | GO:0006897; GO:0005044; GO:0016021 | endocytosis; scavenger receptor activity; integral component of membrane |  |
| 34 | cce1678.5 | negative elongation factor A | GO:0006414; GO:0034244; GO:0003746; GO:0032021 | translational elongation; negative regulation of transcription elongation from RNA polymerase II promoter; translation elongation factor activity; NELF complex |  |
| 35 | cce1712.1 | Cysteine proteinase inhibitor |  |  |  |
| 36 | cce12716.1 | sodium leak channel non-selective protein isoform X1 | GO:0098655; GO:0005261; GO:0016021 | cation transmembrane transport; cation channel activity; integral component of membrane |  |

**TABLE S1.2** continued
